# Supplementary material for: The Impact of Motherhood on Women’s Career Progression: A Scoping Review of Evidence-Based Interventions
Source: Behav Sci (Basel). 2024 Mar 26;14(4):275. doi: 10.3390/bs14040275 (PMC11047346; doi:10.3390/bs14040275)
Supplement: Supplementary file 1 [file behavsci-14-00275-s001.zip › behavsci-2914165-supplementary.pdf]

# Supplementary Tables

**Supplementary Table 1.** *Impact of motherhood on career progression*

| Impact | n | % | Authors |
|---|---|---|---|
| Sacrifice of interests and career aspirations | 13 | 25.0% | Bowyer et al. (2022); Kim et al. (2022); Toyibah (2019); McIntosh et al. (2012); Eren (2022); Clark et al. (2021); El-Far et al. (2021); Borrueco et al. (2023); Miliopoulou & Kapareliotis (2021); Schlehofer (2012); Brown (2010); Herman et al. (2012) |
| Lower salaries | 13 | 25.0% | Bear & Glick (2017); Hardoy et al. (2017); Cha (2014); McIntosh et al. (2012); Frühwirth-Schnatter et al. (2016); Benard & Correll (2010); Hsu (2021); Kelley et al. (2020); Lucifora et al. (2021); Kahn et al. (2014); Abendroth et al. (2014); Magnusson (2010); Samtleben et al. (2019) |
| Difficulty to re-enter academic life or organization after motherhood | 11 | 21.2% | Bowyer et al. (2022); Eren (2022); Nordberg (2019); Hardoy et al. (2017); Halrynjo & Mangset (2022); Reichman & Sterling (2013); Evertsson & Grunow (2012); Mirick & Wladkowski (2020); Borrueco et al. (2023); Samtleben et al. (2019); Herman et al (2012) |
| Career interruptions | 11 | 21.2% | Hardoyet al. (2017); Kim et al. (2022); McIntosh et al. (2012); Frühwirth-Schnatter et al. (2016); Eren (2022); Whittington (2019); Hsu (2021); Evertsson & Grunow (2012); El-Far et al. (2021); Hancioglu & Hartmann (2014); Kinget al. (2023) |
| Lower likelihood of attaining a managerial position | 10 | 19.2% | Kristensen et al. (2017); Hardoy et al. (2017); Ma (2014); King et al. (2023); Härkönen et al. (2016); Maxwell et al. (2018); McIntosh et al. (2012); Whittington (2019); Lucifora et al. (2021); Mirick & Wladkowski (2020); Eren (2022); |

| | | | |
|---|---|---|---|
| Working part-time | 10 | 19.2% | McIntosh et al. (2012); Whittington (2019); Misra et al. (2012); Zheng et al. (2022); Hsu (2021); Abele & Spurk (2011); Lucifora et al. (2021); Brown (2010); Herman et al. (2012); Hancioglu & Hartmann (2014) |
| Negative attitudes towarrd mothers | 8 | 15.4% | Maxwell et al. (2018); Benard & Correll (2010); Schlehofer (2012); Abendroth et al. (2014); Herman et al. (2012); Samtleben et al. (2019); Miliopoulou & Kapareliotis (2021); Thébaud & Taylor (2021) |
| Work-family conflict | 7 | 13.5% | Hardoy et al. (2017); Clark et al. (2021); Ziegler, et al. (2022); Abele & Spurk (2011); Brown (2010); Mirick & Wladkowski (2020); Borrueco et al. (2023) |
| Having to do more than male counterparts to prove themselves | 6 | 11.5% | Bowyer et al. (2022); Toyibah (2019); Eren (2022); Benard & Correll (2010); Luekemann & Abendroth (2018); Miliopoulou & Kapareliotis (2021) |
| Devaluation of women's abilities | 6 | 11.5% | McIntosh et al. (2012); Benard & Correll (2010); Schlehofer (2012); Miliopoulou & Kapareliotis (2021); Thébaud & Taylor (2021); Herman et al. (2012) |
| Stress, guilt and lower sence of control | 4 | 7.79% | Bowyer et al. (2022); Clark et al. (2021); Brown (2010); Miliopoulou & Kapareliotis (2021) |
| Lower likelihood of being recommended for hiring | 3 | 5.8% | Benard, & Correll (2010); Brown (2010); Thébaud & Taylor (2021) |
| Higher return rates after childbirth | 2 | 3.8% | Ma (2014); Magnusson (2010) |
| Lower sense of belonging | 2 | 3.8% | Bowyer et al. (2022); Miliopoulou & Kapareliotis (2021) |
| Lower sense of entitlement to use benefits | 2 | 3.8% | Herman & Lewis (2012); Herman et al. (2012) |
| Non-submission of manuscripts or canceled research projects | 2 | 3.8% | Staniscuaski, et al.(2021); Samtleben et al. (2019) |

| | | | |
|---|---|---|---|
| Lower productivity on remote work | 2 | 3.8% | Staniscuaski et al. (2021); El-Far et al. (2021) |
| Lower satisfaction with work achievements | 2 | 3.8% | Zheng et al. (2022); Kahn et al. (2014) |
| Higher turnover | 2 | 3.8% | Frühwirth-Schnatter et al. (2016); Halrynjo & Mangset (2022) |
| Developed skills and habilities due to motherhood | 1 | 1.9% | Ma et al. (2022) |
| Increased cognitive knowledge and learning | 1 | 1.9% | Ma et al. (2022) |
| Increased mindset, willpower and emotional intelligence | 1 | 1.9% | Ma et al. (2022) |
| Increased confidence to pursue management and leadership roles | 1 | 1.9% | Ma et al. (2022) |
| Enhanced social relationships | 1 | 1.9% | Ma et al. (2022) |
| Positive workplace outcomes | 1 | 1.9% | Ma et al. (2022) |
| Higher motivation to complete coursework and/or dissertation | 1 | 1.9% | Mirick & Wladkowski (2020) |
| Higher perception of mothers as ideal managers than nonmothers | 1 | 1.9% | Morgenroth & Sønderlund (2021) |
| Greater appreciation of mother's work | 1 | 1.9% | Thébaud & Taylor (2021) |
| Increased pressure due to the new responsibilities | 1 | 1.9% | Clark et al. (2021) |
| Pursuit of flexible jobs | 1 | 1.9% | Kim et al. (2022) |
| Failure to meet deadlines | 1 | 1.9% | Staniscuaski et al. (2021). |
| Dropping out | 1 | 1.9% | Eren (2022) |

| | | | |
|---|---|---|---|
| Rejection of research fundings | 1 | 1.9% | Eren (2022) |
| Fear of losing the job | 1 | 1.9% | Borrueco et al. (2023) |
| Discussions about the personal life | 1 | 1.9% | Schlehofer (2012) |
| Time-consuming work is higher in women | 1 | 1.9% | Magnusson (2010) |
| Lower assignment of interesting tasks | 1 | 1.9% | Samtleben et al. (2019) |
| Lower perception of mothers as ideal managers than fathers | 1 | 1.9% | Morgenroth et al. (2021) |
| Negative views about using benefits | 1 | 1.9% | Miliopoulou & Kapareliotis (2021) |

**Supplementary Table 2.** *Career interventions for mothers' career progress*

| Level of analysis (based on Lau et al., 2023) | Intervention | Rationale |
|---|---|---|
| ***Ontogenic*** (individual level, actions focused on individuals, to promote changes in beliefs, attitudes, dispositions or behaviors) | Career counseling and family planning | Helping mothers to make informed career decisions and family planning, while also being aligned with their own values and identity. |
| | Individual counseling and support groups | Addressing identity conflicts, internalized stigma, navigating societal expectations and developing coping strategies to deal with the challenges of being both a mother and a career woman |
| | Education and skills development | Developing skills such as time management and communication to facilitate negotiations for working conditions and the career-family balance. Education on women's rights to protect mothers against discrimination at work and financial literacy education to help them navigate career transitions, especially during economic crisis. |
| ***Interpersonal*** (immediate social context, to change interpersonal relations and foster a supportive and inclusive environment) | Family, friends and community support | Building a support system to share family responsibilities, allowing mothers career advancement |
| | Equal share of family responsibilities with partner | Challenging traditional gender roles through equal distribution of household and caregiving chores between spouses, with clear communication and division of responsibilities. |
| | Role models | Identifying and connecting with successful working mothers to obtain inspiration, guidance and effective strategies to achieve career progress |

| | | |
|---|---|---|
| | Peer support network | Sharing knowledge, experiences or advice with other parents, besides creating a safe space for mutual encouragement and emotional support. |
| | Regular communication and rituals for working mothers | Attenuating feelings of guilt and separation anxiety, besides maintaining a strong connection between working mothers and their families. |
| ***Organizational*** (broader societal context, changes in workplace culture, policies and practices) | Flexible work arrangements and policies | Recognizing the unique challenges faced by mothers by offering flexible working options (e.g. adjusting work hours, remote work options, no late meetings) and temporary role restructuring (e.g. less field work for new parents), in response to changing family circumstances. |
| | Family-friendly benefits | Creating an environment that enables mothers to balance work and family responsibilities, through extended parental leave, on-site childcare facilities and inclusive spaces, events and activities for children. |
| | Replacement arrangements and structured return programs | Temporarily replacing employees on parental leave to ensure the continuity of the workflow, guarantee job security and alleviate the pressure of taking extended leave. Promoting structured return programs to ensure a smooth reintegration of mothers in the workforce (e.g. phased return schedules, mentorship and training). |
| | Anti-discrimination and anti-harassment policies | Protecting working mothers against sexist practices and discrimination by employers, with clear mechanisms for reporting and addressing harassment incidents. |

| | |
|---|---|
| Implicit bias training | Raising awareness of gender stereotypes that may affect the perception of mothers, such as assumptions about commitment, skills or availability, that commonly reflect on decisions about career progression. |
| Transparent salary and promotion practices | Fostering fair compensation based on clear criteria for hiring, salary and promotion, to minimize the impact of subjective interpersonal perceptions. To avoid gender disparities, it is important not to rely solely on productivity criteria and ignore caregiving status, but also to recognize the context, responsibilities and challenges faced by mothers, while also recognizing the skills and experiences gained through caregiving. |
| Collaborative approach to work | Promoting shared demands, responsibilities and rewards, to dismantle traditional hierarchies, promote inclusivity and support mothers' career progression. |
| Review academic criteria for recognition, tenure and promotion | Rethinking the role of productivity in the academic reward system to accommodate mothers diverse career trajectories, by recognizing diverse forms of scholarly output (e.g. data sharing, teaching, collaborative projects and interventions). |
| Career development programs for women | Promoting female talent through mentorship programs, succession planning or leadership programs. It is also important to recognize motherhood as a good opportunity to develop leadership skills and foster collaborative leadership styles, challenging the traditional views of what is a good leader. |

| | | |
|---|---|---|
| | Formal women's network | Creating a structured platform for women to connect, share experiences and support each other to navigate the challenges of balancing work and family responsibilities. This can be a source of inspiration, guidance and establishment of informal mentorship relations. |
| | Supportive work culture | Creating an environment that celebrates work-family balance and cultivates positive attitudes toward working mothers. Besides family-friendly benefits, it involves creating open platforms for discussions about motherhood, inviting mothers to share their experiences and recognizing soft skills developed through child care and making the presence of mothers in the workplace increasingly normative. |
| ***Macrosystem*** (larger social and cultural contexts, target structural changes, such as public policies, legislations or widespread cultural shifts) | Public policies | Creating a family-friendly ecosystem through legislations that provide affordable, reliable and accessible childcare services, offering tax credits for childcare expenses, creating of lactation spaces, providing free public education and comprehensive national parental leave policies. |
| | Education initiatives | Policies and programs designed to provide training and learning resources to mothers, enhancing their employability keep them updated on industry trends and facilitate their return after career breaks. |

| | |
|---|---|
| Awareness programs | Awareness campaigns to educate the public about the stigmas associated with motherhood and challenging traditional gender norms, by highlighting successful stories of working mothers, bringing attention to the systemic barriers and stereotypes faced by them, or communicating the benefits associated with family-friendly policies. |
| Social safety nets and government programs | Offering financial support, healthcare, food security and housing assistance for low income families, added to reemployment programs, especially during economic crisis. These are expected to attenuate the financial expenses associated with children, which are especially concerning for single mothers and those from disadvantaged backgrounds. |
| Labor market regulations for job stability | Protecting the rights of part-time and short-term contract workers, considering this is the career path followed by many women after motherhood, by ensuring job security, fair treatment and mechanisms for transitioning contract workers to permanent roles. |
| Maternity support policies in academia | Changing evaluation metrics in academia, by recognizing diverse research outputs other than publications, provide funding for hiring research assistance, postponing deadlines, provide flexible work arrangements and gender-neutral extended parental leave, besides funding research on gender biases or working mothers experiences to inform future interventions. |

| | |
|---|---|
| Cultural shift | Challenging traditional gender norms about what it means to be a worker and a leader, while redefining societal perceptions of motherhood by celebrating the diversity of roles women can occupy. The cultural shift also needs to involve valuing care work, by recognizing its socioeconomic significance, the effort spent and the skills necessary. |
